# Supplementary material for: Response of soil nematode community structure, diversity, and ecological network to elevation gradients in wild fruit forest of Tianshan Mountain, China
Source: PeerJ. 2025 Oct 1;13:e20090. doi: 10.7717/peerj.20090 (PMC12495954; doi:10.7717/peerj.20090)
Supplement: Supplemental Information 2 [file peerj-13-20090-s002.docx]

# **Supplemental Tables**

**Table S1. Soil Nematode Composition and Quantity at Different Altitudes in Tianshan Wild Fruit Forest, Xinjiang (n/ind. (100 g)^-1^)**

**Table S2.** **Chemical properties of wild fruit forests in the Tianshan Mountains under different altitudinal gradients**

**Table S1. Soil Nematode Composition and Quantity at Different Altitudes in Tianshan Wild Fruit Forest, Xinjiang (n/ind. (100 g)^-1^)**

| Group | Trophic group | NUM | Elevation | | | | | | | | | | | |
| --- | --- | --- | --- | --- | --- | --- | --- | --- | --- | --- | --- | --- | --- | --- |
|  |  |  | JC | | | | | | MS | | | | | |
|  |  |  | E1（1480m） | E2（1401m） | E3（1351m） | E4（1305m） | E5（1252m） | E6  （1207m） | E1（1480m） | E2（1401m） | E3（1351m） | E4（1305m） | E5（1252m） | E6  （1207m） |
| **Bacterial-feeding** |  |  |  |  |  |  |  |  |  |  |  |  |  |  |
| *Acrobeles* | Ba2 | G1 | **180.69** | 43.52 | 88.60 | **189.24** | 54.39 | 18.06 | 56.72 | 86.84 | 84.80 | 93.47 | 84.68 | 40.89 |
| *Acrobeloides* | Ba2 | G2 | 71.03 | 32.74 | 42.57 | 55.62 | 85.49 | 43.91 | 45.43 | 46.16 | 62.96 | **134.05** | 68.95 | 14.22 |
| *Cervidellus* | Ba2 | G3 | 2.95 | 103.00 | 87.65 | 63.73 | 14.59 |  | 63.09 | 43.06 | 36.02 | 80.84 | 7.87 | 9.51 |
| *Prismatolaimus* | Ba3 | G4 |  | 30.96 | 84.97 | 90.61 | 14.25 |  | 67.08 |  | 60.38 | 79.59 |  |  |
| *Plectus* | Ba2 | G5 |  | 47.91 | 12.07 | 69.13 | 1.19 |  | 32.20 | 27.26 | 23.50 | 31.98 |  |  |
| *Panagrolaimus* | Ba1 | G6 | 8.89 | 7.84 | 87.64 | 5.70 | 27.23 | 18.61 | 2.94 | 4.23 | 51.16 | 19.75 |  |  |
| *panagrellus* | Ba1 | G7 |  | 26.10 | 40.75 |  |  |  | 29.06 | 23.81 | 19.25 | 16.59 |  | 4.92 |
| *Chiloplacus* | Ba2 | G8 | 47.46 | 1.96 |  |  |  | 8.12 | 3.81 | 1.24 |  | 3.03 |  | 33.03 |
| *Wilsonema* | Ba2 | G9 |  | 13.87 | 23.19 | 7.07 |  |  | 13.19 | 7.51 | 7.62 | 12.30 |  |  |
| *Rhabditis* | Ba1 | G10 | 11.01 |  |  | 6.11 | 23.62 | 1.76 | 4.24 | 1.28 |  | 2.62 |  | 5.51 |
| *Cephalobus* | Ba2 | G11 | 1.48 | 4.94 |  | 1.51 | 10.09 | 5.40 | 1.34 | 2.98 | 13.20 | 1.36 |  | 1.19 |
| *Eucephalobus* | Ba2 | G12 |  |  | 2.25 | 1.66 |  | 4.70 | 3.17 |  | 2.06 |  |  | 3.17 |
| *Anaplectus* | Ba2 | G13 |  |  |  |  | 7.29 |  |  |  |  |  |  |  |
| *Pelodera* | Ba1 | G14 | 1.48 | 1.18 | 1.34 |  |  |  | 2.77 |  |  |  |  |  |
| *Bastiania* | Ba3 | G15 |  |  |  |  |  |  | 1.42 |  | 3.59 |  |  |  |
| *Teratocephalus* | Ba3 | G16 |  | 1.56 |  |  |  |  |  |  | 1.53 |  |  |  |
| *Monhystera* | Ba2 | G17 |  |  |  | 1.57 |  |  |  |  |  |  | 1.27 |  |
| *Geomonhystera* | Ba2 | G18 |  | 1.37 |  |  |  |  |  |  |  |  |  |  |
| *Odontolaimus* | Ba3 | G19 |  |  |  |  |  | 1.23 |  |  |  |  |  |  |
| *Diploscapter* | Ba1 | G20 | 0.61 |  |  |  |  |  |  |  |  |  |  |  |
| Group | Trophic group | NUM | Elevation | | | | | | | | | | | |
|  |  |  | JC | | | | | | MS | | | | | |
|  |  |  | E1（1480m） | E2（1401m） | E3（1351m） | E4（1305m） | E5（1252m） | E6（1207m） | E1（1480m） | E2（1401m） | E3（1351m） | E4（1305m） | E5（1252m） | E6  （1207m） |
| **Fungal-feeding** |  |  |  |  |  |  |  |  |  |  |  |  |  |  |
| *Aphelenchus* | Fu2 | G21 | 69.72 | 111.27 | 73.58 | 68.35 | 63.96 | 63.26 | 57.86 | 52.30 | 94.49 | 44.22 | 27.91 | 55.49 |
| *Paraphelenchus* | Fu2 | G22 |  | 50.02 | 95.37 | 35.46 | 38.04 | 16.73 | 17.16 | 58.58 | 79.94 | 15.97 | 20.20 | 11.43 |
| *Aphelenchoides* | Fu2 | G23 | 10.62 | 17.52 | 21.60 | 3.27 | 33.86 | 20.15 | 21.44 | 35.43 | 44.38 | 6.72 | 11.82 | 10.98 |
| *Tylencholaimus* | Fu4 | G24 |  | 22.61 | 8.79 | 17.15 |  |  | 13.75 | 7.26 | 11.09 | 4.39 |  |  |
| *Diphtherophora* | Fu3 | G25 |  | 6.93 | 19.19 | 5.70 |  |  | 4.24 | 15.29 | 2.93 | 23.53 |  |  |
| *Dotylaphus* | Fu2 | G26 |  |  | 33.54 |  |  |  |  |  | 1.53 |  |  |  |
| *Dorylaimoides* | Fu4 | G27 |  |  | 3.08 |  |  |  | 2.73 | 6.09 | 2.21 | 9.00 |  |  |
| *Ditylenchus* | Fu2 | G28 | 5.04 | 1.18 |  |  |  | 1.30 | 1.18 |  |  | 1.60 | 1.27 | 10.22 |
| *Tylencholaimellus* | Fu4 | G29 |  |  |  | 6.60 |  | 1.76 | 1.48 | 3.57 |  |  |  |  |
| *Campydora* | Fu4 | G30 |  |  |  |  |  | 1.76 | 4.24 | 5.04 | 1.46 |  |  |  |
| *Fungiotonchium* | Fu2 | G31 |  | 1.30 |  |  |  |  |  |  |  |  |  |  |
| **Plant-feeding** | |  |  |  |  |  |  |  |  |  |  |  |  |  |
| *Paratylenchus* | Pl2 | G32 | **180.99** | **135.92** | **163.47** | **275.44** | **277.64** | **53.60** | 84.98 | **117.29** | **228.81** | **82.65** | **261.47** | **107.24** |
| *Merlinius* | Pl3 | G33 | 104.52 | 112.07 | 45.31 | 219.31 | 47.37 | 31.68 | 19.21 | 48.84 | 97.16 | 32.87 | 95.13 | 46.03 |
| *Coslenchus* | Pl2 | G34 | 119.15 | 57.43 | 92.51 | 58.56 | 60.94 | 19.60 | 44.72 | **117.82** | 23.39 | 69.08 | 44.41 | 70.07 |
| *Basiria* | Pl2 | G35 | 17.49 | 25.00 | 28.16 | 46.27 | 130.96 | 51.74 | 25.49 | **151.77** | 14.39 | 45.84 | 133.79 | 18.01 |
| *Helicotylenchus* | Pl3 | G36 | 5.79 | 70.24 | 87.09 | 7.27 | 29.43 | 47.75 | 12.33 | 10.67 | 69.27 | **177.59** | 7.83 | **95.57** |
| *Malenchus* | Pl2 | G37 | 18.73 | 63.18 | 20.69 | 49.55 | 23.13 | 15.51 | 63.31 | 44.19 | 7.04 | 61.67 | 35.73 | 16.19 |
| *Boleodorus* | Pl2 | G38 | 43.30 | 9.59 | 42.63 | 32.18 | 15.85 | 3.65 | 63.47 | 20.65 | 36.28 | 74.75 | 34.89 | 36.80 |
| *Criconemella* | Pl3 | G39 | 60.51 | 5.93 | 1.22 | 3.15 | 10.46 | 19.84 | 52.23 | 3.66 | 4.11 |  | 78.86 | 81.82 |
| *Rotylenchus* | Pl3 | G40 | 3.54 | 49.96 | 27.57 |  | 28.09 | 10.93 | 8.55 | 6.78 | 39.91 | 38.23 |  | 89.48 |
| Group | Trophic group | NUM | Elevation | | | | | | | | | | | |
|  |  |  | JC | | | | | | MS | | | | | |
|  |  |  | E1（1480m） | E2（1401m） | E3（1351m） | E4（1305m） | E5（1252m） | E6（1207m） | E1（1480m） | E2（1401m） | E3（1351m） | E4（1305m） | E5（1252m） | E6  （1207m） |
| *Filenchus* | Pl2 | G41 |  | 23.13 | 7.80 | 72.71 | 7.11 | 1.17 | 36.27 | 31.43 | 5.14 | 34.64 | 43.51 | 9.89 |
| *Macroposthonia* | Pl3 | G42 |  | 1.60 |  |  | 26.61 |  | 98.36 |  |  |  | 123.73 | 1.25 |
| *Pararotylenchus* | Pl3 | G43 | 12.84 | 40.43 | 9.95 |  |  | 2.69 | 13.96 | 6.85 | 39.47 | 48.65 |  | 45.62 |
| *Ogma* | Pl3 | G44 |  | 50.22 | 11.21 | 2.90 | 28.31 |  | 7.95 | 15.48 | 12.34 | 15.40 |  | 4.54 |
| *Nagelus* | Pl3 | G45 | 3.46 | 2.74 |  | 9.40 | 2.86 | 20.97 | 10.93 | 11.47 |  |  | 36.78 | 13.75 |
| *Quinisulcius* | Pl3 | G46 |  | 18.91 | 11.96 | 17.87 |  | 3.99 | 8.79 |  | 21.85 |  | 6.94 | 3.56 |
| *Tylencholaimus* | Pl3 | G47 |  | 10.42 | 10.75 | 8.10 | 1.23 | 2.59 | 1.59 | 2.62 | 8.36 |  |  | 7.68 |
| *Rotylenchulus* | Pl3 | G48 | 7.16 | 8.73 | 4.22 | 5.10 | 5.23 | 1.51 |  |  | 8.19 |  |  | 9.57 |
| *Criconemoides* | Pl3 | G49 | 0.70 | 1.96 | 1.59 |  |  | 19.15 | 5.21 |  |  |  | 10.32 | 4.54 |
| *Tylenchus* | Pl2 | G50 |  |  | 4.50 | 4.00 |  | 5.16 | 7.70 | 2.43 |  | 6.99 |  | 3.10 |
| *Hemicycliophora* | Pl3 | G51 | 0.70 |  | 4.67 | 1.51 | 1.43 | 1.17 |  |  |  | 4.96 |  | 10.53 |
| *Hemicriconemoides* | Pl3 | G52 | 0.70 | 12.22 |  |  |  |  |  |  | 8.23 |  |  |  |
| *Pratylenchus* | Pl3 | G53 |  |  | 5.33 | 3.08 |  |  | 11.35 |  |  |  |  |  |
| *Neothada* | Pl2 | G54 | 7.70 |  | 2.25 |  |  |  | 1.48 | 4.96 |  |  |  |  |
| *Lelenchus* | Pl2 | G55 | 1.47 |  |  |  |  | 2.35 |  | 1.26 |  |  |  | 6.39 |
| *Geocenamus* | Pl3 | G56 |  |  |  |  |  |  |  | 5.14 |  |  |  | 6.15 |
| *Aglenchus* | Pl2 | G57 |  |  |  | 4.85 |  | 1.30 |  |  | 1.46 |  | 1.59 |  |
| *Crossonema* | Pl3 | G58 |  | 1.37 |  |  |  |  |  | 4.76 |  |  |  |  |
| *Cephalenchus* | Pl2 | G59 |  |  |  | 3.40 |  |  |  |  |  |  |  |  |
| *Dolichorhynchus* | Pl3 | G60 |  |  |  | 3.01 |  |  |  |  |  |  |  |  |
| *Amplimerlinius* | Pl3 | G61 |  |  |  |  | 1.46 |  |  |  |  |  |  | 1.23 |
| *Paratrichodorus* | Pl4 | G62 |  | 1.74 |  |  |  |  |  |  |  |  |  |  |
| Group | Trophic group | NUM | Elevation | | | | | | | | | | | |
|  |  |  | JC | | | | | | MS | | | | | |
|  |  |  | E1（1480m） | E2（1401m） | E3（1351m） | E4（1305m） | E5（1252m） | E6（1207m） | E1（1480m） | E2（1401m） | E3（1351m） | E4（1305m） | E5（1252m） | E6  （1207m） |
| *Heterodera* | Pl3 | G63 |  |  | 1.49 |  |  |  |  |  |  |  |  |  |
| **Predators-Omnivores** | |  |  |  |  |  |  |  |  |  |  |  |  |  |
| *Discolaimium* | Pr5 | G64 | 11.51 | 8.17 | 11.72 | 14.99 | 23.99 | 23.64 | 4.09 | 8.96 | 32.99 | 5.66 | 3.19 | 3.83 |
| *Dorylaimellus* | Pr5 | G65 |  | 29.55 | 6.47 | 23.46 | 1.43 |  | 13.03 | 16.60 | 12.16 | 7.53 |  | 6.68 |
| *Mylonchulus* | Pr4 | G66 |  |  | 2.25 | 17.22 | 65.60 |  |  |  |  | 10.44 |  | 1.59 |
| *Microdorylaimus* | Pr4 | G67 | 8.60 |  | 6.39 | 2.30 | 10.23 | 4.81 | 7.28 | 1.20 | 25.08 |  | 2.57 |  |
| *Pungentus* | Pr4 | G68 | 3.42 |  |  |  | 8.22 | 13.15 |  |  | 2.06 | 2.96 | 35.08 | 1.34 |
| *Paraxonchium* | Pr5 | G69 |  | 1.60 | 11.25 | 4.90 | 11.63 | 1.76 | 2.68 | 4.97 | 5.88 | 4.02 | 3.62 |  |
| *Thonus* | Pr4 | G70 |  | 2.03 | 1.22 | 1.70 |  |  | 23.98 | 1.38 | 1.55 |  |  |  |
| *Eudorylaimus* | Pr4 | G71 |  |  |  |  |  | 2.99 |  |  |  |  | 23.47 | 2.37 |
| *Torumanawa* | Pr5 | G72 |  |  | 4.66 | 1.45 | 1.43 |  | 6.71 | 2.57 | 1.46 | 4.51 |  | 3.17 |
| *Monochromadora* | Pr3 | G73 | 6.67 | 1.18 | 4.66 |  | 2.37 | 1.30 | 3.17 |  |  |  |  | 1.33 |
| *Discolaimus* | Pr5 | G74 |  |  |  |  |  |  | 7.06 | 4.24 |  | 1.67 |  | 1.19 |
| *Mesodorylaimus* | Pr5 | G75 |  |  |  |  | 1.43 |  | 2.94 |  |  | 1.74 |  | 5.01 |
| *Parkellus* | Pr4 | G76 |  |  |  |  | 1.43 | 7.46 |  |  |  |  | 1.30 |  |
| *Dorydorella* | Pr4 | G77 |  |  | 1.89 |  |  | 1.76 |  |  |  | 1.40 |  |  |
| *Laimydorus* | Pr5 | G78 | 1.48 |  |  |  |  | 3.03 |  |  |  |  |  |  |
| *Axonchium* | Pr5 | G79 |  |  |  |  | 1.46 |  | 1.48 |  |  |  |  | 1.33 |
| *Sectonema* | Pr5 | G80 |  |  |  |  |  |  |  |  |  | 1.74 |  |  |
| Total individuals | |  | 501.29 | 687.89 | 542.37 | 835.10 | 766.38 | 356.64 | 605.60 | 530.17 | 683.21 | 665.92 | 939.80 | 646.77 |
| Total group number |  |  | 21 | 25 | 29 | 26 | 27 | 28 | 29 | 25 | 23 | 22 | 19 | 32 |

Note: The dominant taxa are shown in bold. Dominant genera >10 %; common genera, 1% ~ 10%; rare genera < 1%.

**Table S2.** **Chemical properties of wild fruit forests in the Tianshan Mountains under different altitudinal gradients**

| Habitat | Altitude | Soil organic carbon  (g/kg) | Total nitrogen  (g/kg) | Total phosphorus  (g/kg) | Total potassium  (g/kg) | Nitrate nitrogen  (mg/kg) | Ammonium nitrogen  (mg/kg) | Available potassium  (mg/kg) | Available phosphorus  (mg/kg) | pH | Electrical  conductivity | Soil moisture  Content (%) |
| --- | --- | --- | --- | --- | --- | --- | --- | --- | --- | --- | --- | --- |
| *Juglans cathayensis*  forest | 1480m | 88.03 | 7.75 | 0.18 | 19.84 | 1.30 | 9.81 | 874.13 | 16.33 | 7.92 | 250.00 | 0.37 |
|  | 1401m | 122.70 | 10.73 | 0.17 | 19.88 | 19.36 | 19.35 | 925.97 | 12.84 | 6.64 | 162.00 | 0.44 |
|  | 1351m | 113.48 | 13.76 | 0.19 | 19.72 | 30.91 | 23.44 | 905.96 | 14.17 | 7.29 | 205.00 | 0.49 |
|  | 1305m | 108.78 | 11.15 | 0.19 | 19.40 | 20.62 | 18.94 | 753.73 | 16.92 | 6.66 | 141.00 | 0.48 |
|  | 1252m | 103.38 | 15.13 | 0.23 | 19.71 | 33.99 | 15.65 | 869.05 | 36.86 | 7.62 | 276.00 | 0.43 |
|  | 1207m | 85.89 | 7.88 | 0.22 | 20.64 | 18.48 | 11.20 | 766.03 | 16.71 | 6.81 | 170.00 | 0.32 |
| *Malus sieversii*  forest | 1480m | 78.01 | 7.78 | 0.16 | 20.07 | 13.19 | 15.80 | 694.42 | 11.16 | 7.65 | 178.00 | 0.32 |
|  | 1401m | 87.14 | 7.69 | 0.19 | 21.04 | 2.13 | 4.20 | 747.31 | 10.30 | 6.99 | 65.00 | 0.28 |
|  | 1351m | 85.56 | 8.45 | 0.21 | 21.23 | 37.50 | 16.28 | 1186.96 | 15.21 | 6.85 | 222.00 | 0.38 |
|  | 1305m | 114.55 | 12.61 | 0.20 | 19.52 | 41.87 | 21.06 | 781.35 | 19.01 | 6.63 | 217.00 | 0.43 |
|  | 1252m | 112.77 | 12.63 | 0.23 | 19.49 | 19.15 | 16.67 | 895.95 | 20.33 | 7.38 | 215.00 | 0.41 |
|  | 1207m | 85.39 | 10.85 | 0.22 | 20.85 | 17.50 | 14.61 | 895.46 | 17.32 | 7.76 | 244.00 | 0.29 |
